# *Wolbachia*-Host Interactions: Host Mating Patterns Affect *Wolbachia* Density Dynamics

**DOI:** 10.1371/journal.pone.0066373

**Published:** 2013-06-18

**Authors:** Dong-Xiao Zhao, Xiang-Fei Zhang, Da-Song Chen, Yan-Kai Zhang, Xiao-Yue Hong

**Affiliations:** Department of Entomology, Nanjing Agricultural University, Nanjing, Jiangsu, China; Emory University, United States of America

## Abstract

*Wolbachia* are maternally inherited intracellular bacteria that infect a wide range of arthropods and cause an array of effects on host reproduction, fitness and mating behavior. Although our understanding of the *Wolbachia*-associated effects on hosts is rapidly expanding, our knowledge of the host factors that mediate *Wolbachia* dynamics is rudimentary. Here, we explore the interactions between *Wolbachia* and its host, the two-spotted spider mite *Tetranychus urticae* Koch. Our results indicate that *Wolbachia* induces strong cytoplasmic incompatibility (CI), increases host fecundity, but has no effects on the longevity of females and the mating competitiveness of males in *T. urticae*. Most importantly, host mating pattern was found to affect *Wolbachia* density dynamics during host aging. Mating of an uninfected mite of either sex with an infected mite attenuates the *Wolbachia* density in the infected mite. According to the results of *Wolbachia* localization, this finding may be associated with the tropism of *Wolbachia* for the reproductive tissue in adult spider mites. Our findings describe a new interaction between *Wolbachia* and their hosts.

## Introduction

Endosymbiotic bacteria are very common in invertebrates [1]. The α-proteobacterium *Wolbachia pipientis*, one of the most common endosymbionts, infects an estimated 25–76% of all insect species, as well as many other arthropod and filarial nematode species [2], [3], [4]. *Wolbachia* have a remarkable variety of effects on host biology, including reproduction, fitness and mating behavior.

Vertically inherited by transovarial transmission, *Wolbachia* have evolved a number of different manipulations of host reproduction which impart a selective advantage for the bacteria [5], [6]. Within arthropods, these manipulations include feminization, male killing, parthenogenesis, and cytoplasmic incompatibility (CI), the inability of infected males to successfully fertilize eggs from uninfected females [7]. Cytological analyses suggest that *Wolbachia* target cell cycle regulatory proteins. CI results from delayed nuclear envelope breakdown of the male pronucleus [8]. CI has been described in most insect orders (Diptera, Coleoptera, Hymenoptera, Orthoptera, and Lepidoptera) [7], in mites [9], [10], [11], [12], and in the terrestrial isopod *Porcellio dilatatus* [13]. Although most molecular mechanisms employed by *Wolbachia* to manipulate the host cytoplasmic machinery and to ensure vertical transmission have not been discovered, intensive research in host-*Wolbachia* interactions and genomes of *Wolbachia* strains have provided important hints to reveal the molecular mechanism [14].

The interaction between *Wolbachia* and its hosts can evolve rapidly over time [15], leading to both positive and negative effects on host fitness, and even to the extreme case where *Wolbachia* become essential for host fertility. In some cases, such as in the parasitoid wasp *Leptopilina heterotoma*, *Wolbachia* can negatively affect fecundity, locomotor performance and longevity [16]. On the other hand, super *Wolbachia* infection (co-infection with two or more *Wolbachia* strains) has been reported to improve fecundity in *Aedes albopictus*, accelerating the rate of *Wolbachia* invasion in the host population [17]. Furthermore, oogenesis of the parasitic wasp *Asobara tabida* is dependent on *Wolbachia* [18].

One of the most interesting discoveries about *Wolbachia* infection is that infected *Drosophila simulans* males produce less sperm than their uninfected counterparts [19]. This led to the hypothesis that *Wolbachia* infection affects the mating behavior of males as a consequence of this physiological cost. Several reports have provided support for this hypothesis. Champion *et al*. examined the impact of *Wolbachia* on mating behavior in male *Drosophila melanogaster* and *D. simulans*, and showed that infected males mate at a higher rate than uninfected males in both species [20]. In addition, male *D. simulans* exhibited some preference for mating with females of the same infection status [21]. More importantly, *Wolbachia* promote speciation of *Drosophila melanogaster* by contributing to the level of mate discrimination between diverging *D. melanogaster* populations [22].

Although it is clear that *Wolbachia* affect the reproduction and behavior of hosts, do hosts also affect the dynamics of *Wolbachia*? We used the two-spotted spider mite –*Wolbachia* symbiosis to address this question. The two-spotted spider mite *Tetranychus urticae* Koch is a worldwide pest threatening many agricultural crops and fruit trees. We previously showed that *Wolbachia* was widely distributed in Chinese populations of *T. urticae*. All populations were found to be infected with *Wolbachia*, with the infection rate ranging from 2.5 to 85% [23]. Several studies have shown that *Wolbachia* can induce variable reproduction and fitness effects on the two-spotted spider mite *T. urticae* [9], [24], [25], [26]. *Wolbachia* can also affect oviposition and mating behavior of *T. urticae* [27]. In this study, we explored the *Wolbachia*-effects on the spider mite host by measuring the strength of CI, sex ratio, fecundity, survival and male mating competitiveness between infected and uninfected strains under laboratory conditions. In order to improve our understanding of interactions between *Wolbachia* and the two-spotted spider mite *T. urticae*, we also determined whether the mite influences *Wolbachia* dynamics by examining the relative density of *Wolbachia* in mites whose mates had different infection statuses. In addition, we studied *Wolbachia* spatial localization in the adults using fluorescence in situ hybridization (FISH).

## Materials and Methods

### Preparation of Spider Mite Lines

#### Ethics statement

No specific permits were required for the described field studies. (a) No specific permissions were required for the collection because the spider mite is a pest on the soybean; (b) The location is not privately-owned in any way; (c) The field studies did not involve endangered or protected species.

The two-spotted spider mite *T. urticae* was collected from soybean [*Glycine max* (L.) Merr.] leaves in Hohhot, Inner Mongolia, northeast China in July 2010. Mites were reared on a leaf of the common bean (*Phaseolus vulgaris* L.) placed on a water-saturated sponge mat in Petri dishes (dia. 9) at 25±1°C, 60% r.h. and under L16-D8 conditions.

To evaluate the effects of *Wolbachia* on spider mites, 100% infected and 100% uninfected lines were prepared. Females from the teleiochrysalis stage were allowed to lay eggs without being crossed with males. The eggs were reared until adulthood (males). After the males reached sexual maturity, they were backcrossed with the mothers. Then, the female adults were transferred to new leaf discs and were allowed to lay eggs for 3–5 days. The females were checked for *Wolbachia* infection status by PCR amplification. The eggs were separately reared on new leaf discs depending on the infection status of the mother. The above process was continued for three to four generations until a 100% infected population was obtained. The eggs of the uninfected mothers were reared to establish the uninfected line.

### DNA Extraction and Diagnostic PCR

DNA was extracted by homogenizing a single adult in a 25 µl mixture of sodium chloride-Tris-EDTA (STE) buffer (100 mM NaCl, 10 mM Tris-HCl, 1 mM EDTA, pH 8.0) and proteinase K (10 mg/ml, 2 µl) in a 1.5 ml Eppendorf tube. The mixture was incubated at 37°C for 30 min and later 95°C for 5 min. The samples were centrifuged briefly, and used immediately for the PCR reactions or stored at −20°C for later use.

A fragment of the gene encoding the *Wolbachia* surface protein *wsp* was amplified by PCR from samples using primers *wsp*81F and *wsp*691R [28]. We also amplified five *Wolbachia* housekeeping genes for multi-locus sequence typing (MLST) analysis to determine the number of *Wolbachia* strains in this spider mite population [29]. PCR reactions were run in 25 µl buffer using the TAKARA (Takara, Shuzo, Otsu, Japan) Taq kit: 16.3 µl H_2_O, 2.5 µl 10×buffer, 1.5 µl of 2.5 mM deoxyribonucleotide triphosephates (dNTPs), 1.5 µl of 25 mM MgCl_2_, 0.2 µl Taq polymerase (5 U/µl, Takara), 2 µl sample and 1 µl primers (10 mM each). Cycling conditions were 94°C for 2 min followed by 40 cycles of 94°C for 30 s, 52°C for 45 s and 72°C for 1 min and finally 72°C for 7 min. For samples failing to amplify *Wolbachia* genes, primers COI-forward and COI-reverse [30] were used to amplify mitochondria DNA as a positive control for template DNA quality.

Amplified fragments were purified using a Gel Extraction Mini kit (Watson, Shanghai, China). Then, the distinct single-band amplicons were cloned into pGEM T-Easy Vector (Promega, Madison, WI, USA) and the positive clones were screened and finally confirmed by direct sequencing.

### Cross Experiments

In order to determine reproductive compatibility in intra-population crosses, four cross combinations were carried out: uninfected females were crossed with uninfected males, uninfected females were crossed with infected males, infected females were crossed with uninfected males, and infected females were crossed with infected males. Female teleiochrysalids, the last developmental stage before adult emergence, were placed with two males on the same leaf disk. We used 1-day-old virgin males produced as a cohort by groups of females isolated as teleiochrysalids. This procedure was designed to avoid the potential decrease of the *Wolbachia* effect due to male ageing or repeated consecutive mating. Males were discarded 2 days after the females’ eclosion, and mated females were allowed to oviposit for 5 days. Eggs on leaf discs were checked daily to determine the hatchability, sex ratio (% daughters) and mortality of offspring. The effects of infection on female fecundity were tested by comparing the number of eggs laid in the first 5 days by infected and uninfected females who were crossed with uninfected males (♀u×♂u and ♀*w*×♂u) to exclude any influence of differences in male fertility due to infection. To normalize the data, log transformation was used for the number of eggs laid per female, and arcsine square root transformation was used for egg hatchability, sex ratio and mortality. The transformed data which were normally distributed (Kolmogorov-Smirnov test) were analyzed with one-way analysis of variance (ANOVA), and the means were compared using Tukey-HSD test (SPSS 17.0).

### Survival Assessment

Age-specific survival of infected and uninfected lines was measured. Survivorship in infected and uninfected strains was measured by placing a total of 25 virgin females and 25 virgin males of the same infection status on the same leaf. Three leaves were used for each *Wolbachia* infection status. Adult females were monitored at 24-h intervals, during which dead mites were removed and counted. The test was stopped when all the mites had died.

Survival curves for infected and uninfected females were compared using the Kaplan-Meier method [31] and log-rank test [32] (SPSS 17.0).

### Male Mating Competitiveness

In order to compare the mating competitiveness of uninfected and infected males, cross experiments were designed according to the method of Calvitti M *et al* [33]. Three groups of crosses were carried out, including compatible cross (cross A, between uninfected males and females), incompatible cross (cross B, between uninfected females and infected males), and competition study (cross C, between uninfected females and both infected and uninfected males). The competition study was performed by keeping uninfected females and both infected and uninfected males (♀u×[♂ u+♂ *w*]) on the same leaf disk. Four replicates of each cross were performed. Female teleiochrysalids, the last developmental stage before adult emergence, were isolated to assure virginity. Newly emerged females and males were kept on the same leaf disk for 3 days to mate. Females were added after all of the males had been put on the leaf because of the males’ high escape frequency. After oviposition, all females were put on the leaf disks separately. The eggs that hatch successfully are laid by fertile females (mating with uninfected males). By contrast, the eggs that fail to hatch are laid by incompatible females (mating with infected males). In *T. urticae*, females may mate multiply but there is no evidence that they can select sperm once inseminated [34]. Therefore, mating competitiveness between infected and uninfected males can be assessed by comparing the number of compatible and incompatible females. If there were no difference in mating competitiveness between infected and uninfected males, the number of compatible females and incompatible females would be approximately equal.

### Quantitative PCR

Quantitative PCR (Q-PCR) was performed using an ABI PRISM 7300 Sequence Detection System (Applied Biosystems) to estimate the density of *Wolbachia* in *T. urticae*. The 20 µl reaction mixture consisted of 10 µl 2×SYBRq Premix Ex Taq (Takara, Shuzo, Otsu, Japan), 0.4 µl 10 mM of each primer, 0.4 µl 50×ROX Reference Dye, 2 µl DNA template and 6.8 µl H_2_O in single wells of a 96-well plate (PE Applied Biosystems). The Q-PCR cycling conditions included 1 cycle (10 s 95°C) followed by 40 cycles (5 s 95°C, 31 s 60°C), and finally 1 cycle (15 s 95°C, 1 min 60°C, 15 s 95°C). *Wolbachia wsp* gene was quantified using the primer set QwspF (5′-GCA GCG TAT GTA AGC AAT CC-3′) and QwspR (5′-ATA ACG AGC ACC AGC ATA AAG-3′), which amplified a *wsp* fragment (137 bp). To estimate the *Wolbachia* densities, the host the β-actin gene was quantified using the primer set QactinF (5′-CCA TTG AAT CCA AAA GCT AAC CGT-3′) and QactinR (5′-CCA TCA CCA GAG TCG AGG ACA-3′) in the same samples, which amplified a β-actin fragment (149 bp). We used absolute quantitative PCR to determine *wsp* and β-actin copy numbers. Standard curves were plotted using a 10-fold dilution series consisting of 10^−7^ to 10^−3^ dilutions of the DNA standards prepared from plasmid DNA. The quality and concentration of all purified standard DNA were measured by OD absorbance at 260 nm. The copy numbers of each *wsp* and β-actin genes were calculated from the intensity of the fluorescence on the basis of the standard curves. *Wolbachia* density was expressed in terms of the number of *wsp* copies per β-actin copy. *Wolbachia* densities of mites which were virginal, mated to infected ones, and mated to uninfected ones were measured. In order to obtain mites of different mating patterns, newly emerged infected females and males were kept separately and divided into three groups. In the first group, they were kept alone to obtain virgin males and females. In the second group, they were kept with the infected mates for 24 hours. Similarly, in the third group, they were kept with the uninfected mates for 24 hours. All measurements of *Wolbachia* densities in females and males were taken from five ages (2 d, 4 d, 6 d, 8 d, and 10 d) of adults. We compared *Wolbachia* densities in mites of different mating patterns at each age, in order to examine the relationship between the host mating pattern and the density dynamics of *Wolbachia*. DNA of single mites was extracted using the above method. Three replicates were run and averaged for each DNA sample. Negative controls were included in all amplification reactions.

To compare *Wolbachia* densities in mites of different mating patterns, we analyzed data of each age with one-way analysis of variance (ANOVA) and means were compared using the Tukey-HSD test (SPSS17.0).

### Fluorescence in situ Hybridization (FISH)

The fluorescence in situ hybridization (FISH) procedure generally followed the method of Yuval Gottlieb *et al* [35], with slight modifications. Adults were collected with a brush pen under a Nikon SMZ800 stereoscopic microscope (Nikon, Japan). After being washed in 50 µl of phosphate buffered saline (PBS: 8 g NaCl, 0.2 g KCl, and 1.44 g Na_2_HPO_4_ in 1 liter of distilled water, pH 7.2), specimens were put directly into Carnoy’s fixative (chloroform: ethanol: glacial acetic acid, 6∶3∶1) and fixed overnight. After fixation, the samples were decolorized in 6% H_2_O_2_ in ethanol for two hours. Hybridization was performed at 45°C in a dark moisture chamber. After a 30 min pre-incubation period in hybridization buffer (0.9 M NaCl/20 mM Tris·HCl/5 mM EDTA/0.1% SDS/10×Denhardt’s solution), probes were added and incubation was continued for 3 hr. Post-hybridization washes were performed 5 min with 2×SSC pH 7.0 at 45°C, 5 min with 1×SSC pH 7.0 and 5 min with 0.5×SSC pH 7.0 at 37°C, all with agitation. In the end, the adults were put on clean slides (cleaned with alcohol) and were mounted in Vectashield medium (Vector Laboratories). Specific oligonucleotide probes were designed by sequence alignment of *Wolbachia* 16 S rDNA. Two *Wolbachia* probes 5′ end labeled with rhodamine described by Heddi *et al* [36] were used to increase the signals: W1, 5′-AATCCGGCCGARCCGACCC-3′, and W2, 5′-CTTCTGTGAGTACCGTCATTATC-3′. Stained and mounted samples were viewed under a ZEISS LSM 700 confocal microscope (Carl Zeiss, Germany). *Wolbachia* appear as fluorescent red spots. Specificity of the detection was confirmed using the *Wolbachia*-free mites as a control.

## Results

### Multi-locus Sequence Typing (MLST) for *Wolbachia* Infecting *T. urticae*

We amplified the *wsp* gene and a set of housekeeping genes, including *coxA*, *fbpA*, *gatB*, *ftsZ* and *hcpA*, from *Wolbachia* infecting *T. urticae.* The sequences were submitted to the GenBank database (GenBank numbers: *wsp*: JX094384; *coxA*: JX094415; *fbpA*: JX094395; *gatB*: JX094426; *ftsZ*: JX094402; *hcpA*: JX094409). The MLST results show that there is only one strain of *Wolbachia* belonging to supergroup B in this spider mite population [28].

### Strength of Cytoplasmic Incompatibility

*Wolbachia* showed a high level of CI in the *T. urticae* population. The number of aborted eggs was significantly different among the four crosses (Table 1). In the predicted incompatible cross (♀u×♂*w*), on average, 45.0% of all eggs hatched, compared to 93.5–97.3% in the other crosses. The sex ratio of the offspring from the incompatible cross was significantly lower than the sex ratios from the compatible crosses. In addition, the incompatible cross displayed a significantly higher mortality than the compatible crosses.

**Table 1:** Results of crossing experiments between infected (w) and uninfected (u) mites.

Cross type	N	Number of eggs	Hatchability%	Sex ratio (% females)	Daughters	Sons	Total offsprings	Mortality%
♀u×♂u	20	28.35±0.87^b^	94.61±0.72^a^	83.86±0.99a	21.75±0.59a	4.30±0.31	26.05±0.76a	2.72±0.51
♀u×♂*w*	24	30.79±0.71^ab^	45.03±3.19^b^	50.93±3.66b	5.38±0.63b	4.96±0.46	10.33±0.88b	25.57±3.53
♀*w*×♂u	25	33.86±0.64^a^	93.52±0.85^a^	81.01±0.85a	25.00±0.47a	5.86±0.27	30.86±0.47a	2.40±0.46
♀*w*×♂*w*	27	29.41±0.61^b^	97.27±0.91^a^	81.03±1.02a	22.59±0.58a	5.24±0.24	27.82±0.48a	2.54±0.60
		***	***	***	***	NS	***	***

Abbreviations: N, number of replicates; Values for each trait are mean±s.e. NS, not significant;

* *P*<0.05;

** *P*<0.01;

*** *P*<0.001.

Comparisons within a column marked with the same superscript (a, b or c) are not significantly different (*P*>0.05) by a Tukey’s *post hoc* test.

### Effects of *Wolbachia* Infection on Host Fecundity and Longevity

**Figure 1:**
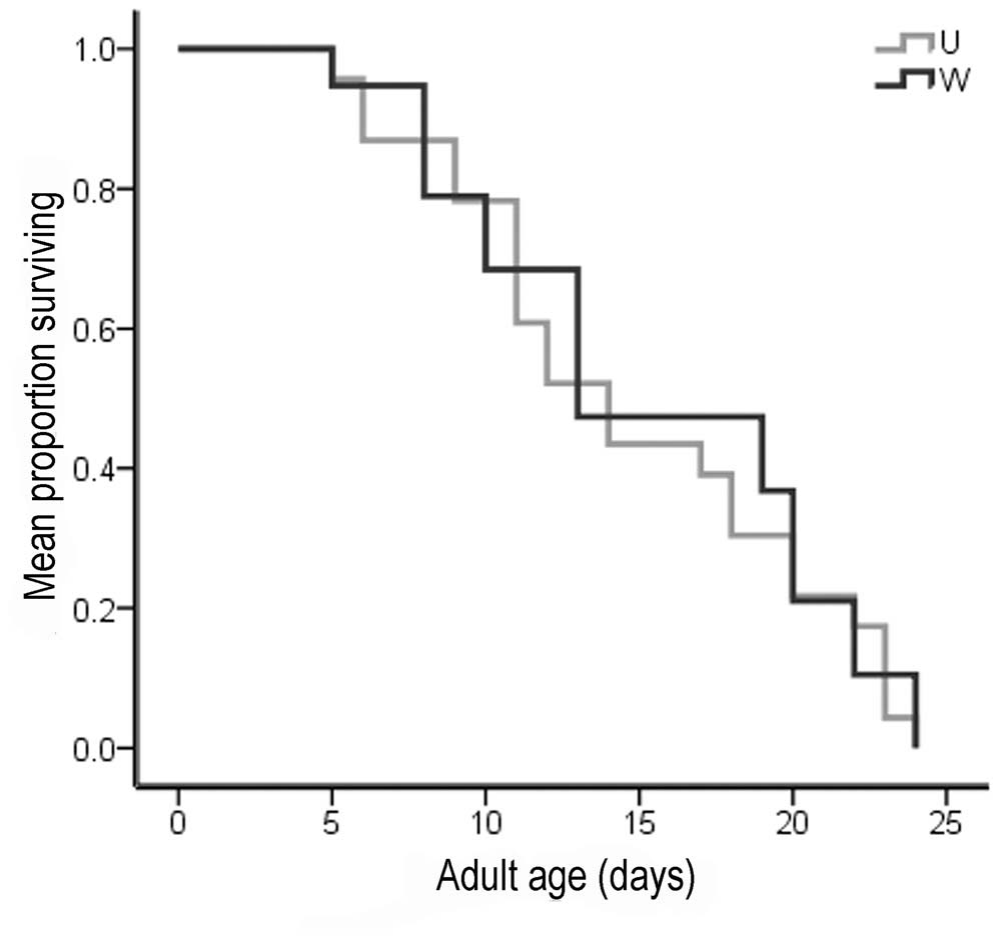
Comparison of *Wolbachia* effect on female longevity in *T. urticae*. W, *Wolbachia* infected strains; U, uninfected strains. Survivor curves for individual hosts were compared using the Kaplan-Meier method and log-rank test.

We compared fecundity of crosses in which females were crossed with uninfected males to exclude any influence of differences in male fertility due to infection. Interestingly, infected females laid significantly more eggs than uninfected females (independent t-test, P<0.001) (Table 1). For infected females, mating with infected males even reduced the number of eggs laid (independent t-test, P<0.001) (Table 1). Survival curves indicated that there was no difference between the mean longevities of infected (15.32±3.27 days) and uninfected females (14.74±1.78 days) (Fig. 1).

### Male Mating Competitiveness

According to the results shown in Table 2, no significant differences in the number of compatible and incompatible females were found in cross C (♀u×[♂u+♂ *w*]) (*X^2^* tests, *P*>0.05), indicating that infected and uninfected males had the same chance to mate with uninfected females. These results indicate that *Wolbachia* has no effects on male mating competitiveness in *T. urticae*.

**Table 2:** Mating competitiveness of *T. urticae* based on the mean number of compatible females and incompatible females.

Cross type		Died	Not inseminated	Inseminated			
				Compatible	Incompatible	*X^2^*	*P*
A. ♀u×♂u	A1	2	0	18	0		
	A2	4	0	16	0		
	A3	1	0	19	0		
	A4	3	0	17	0		
B. ♀u×♂*w*	B1	4	0	0	16		
	B2	3	0	0	17		
	B3	4	0	0	16		
	B4	1	0	0	19		
C. ♀u×(♂u+♂*w*)	C1	3	0	8	9	0.06	*P*>0.05
	C2	2	0	8	10	0.24	*P*>0.05
	C3	1	0	10	9	0.06	*P*>0.05
	C4	4	0	7	9	0.25	*P*>0.05

Abbreviations: Inseminated, females who had daughters (Only male offspring can be produced by parthenogenesis in *T. urticae*); compatible, inseminated females that produced hatching eggs; incompatible, inseminated females that produced non-hatching eggs. *X* ^2^ test, *df*  = 1. *w*, infected strain; u, uninfected strain.

### Wolbachia Density

**Figure 2:**
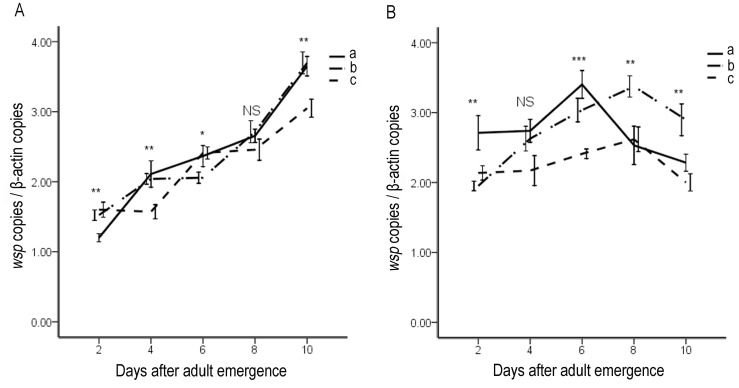
Dynamics of *Wolbachia* density during aging of female (A) and male (B) spider mite *T. urticae* expressed in terms of number of *wsp* copies per β-actin copy. (a) virgin females (males); (b) females (males) mated with infected males (females); (c) females (males) mated with uninfected males (females). Each point is the average of three measurements of 12 samples. Bars indicate standard errors. NS, not significant; *P<0.05; **P<0.01; ***P<0.001.

*Wolbachia* densities (as determined by *wsp* gene relative copy number) in females rose rapidly with age (Fig. 2A). In the virgin females, *Wolbachia* density rose from (1.20±0.06) to (3.65±0.14) step by step from the second day to the tenth day of the mite life span. In addition, *Wolbachia* density increased more rapidly in females mated with infected males (from (1.52±0.07) to (3.69±0.16)) than in females mated with uninfected males (from (1.60±0.10) to (3.04±0.13)). Specifically, from the second day to the tenth day (only except the sixth day), females mated with infected males contain more *Wolbachia* than the females mated with uninfected males. *Wolbachia* replicates most rapidly in virgin females, while it replicates most slowly in females that mated with uninfected males (Fig. 2B). In male adults of *T. urticae*, *Wolbachia* density increased in the younger ones but decreased in the older ones. *Wolbachia* density began to decrease earlier in virgin males than in males that had mated with infected and uninfected females, indicating that mating behavior had a negative effect on the density of *Wolbachia*. Interestingly, *Wolbachia* density in males that mated with uninfected females was comparatively lower than the densities in the other two groups of males (virgin males and males mated with infected females). It also increased very little during the lifespan of the mite. In contrast, mating with infected females was found to be beneficial for the maintenance of *Wolbachia*. During the first 8 days of the males’ life span, the density of *Wolbachia* increased most rapidly. However, the density decreased only slightly from the eighth day to the tenth day. As a result, on the tenth day of the life span, *Wolbachia* density in the males that had mated with infected females was significantly higher than it was in virgin males or males that had mated with uninfected females. Together, these results indicate that mating pattern of hosts affects the dynamics of *Wolbachia* density. Mating with uninfected mites attenuated *Wolbachia* in the infected mites.

### *Wolbachia* Localization

**Figure 3:**
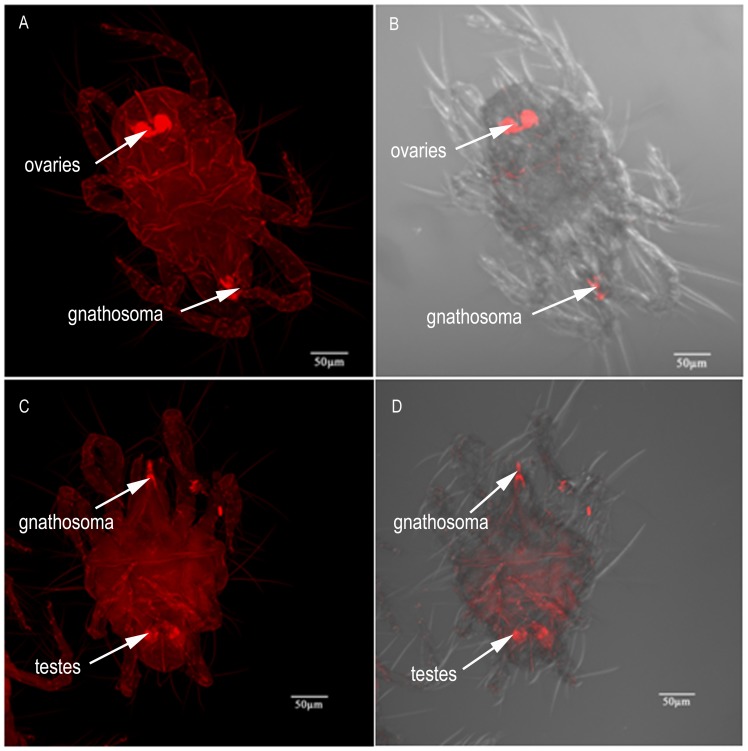
FISH of *T. urticae* adults. (A, B) *Wolbachia* (arrow) in a female abdomen and gnathosoma (combined Z sections). (C, D) *Wolbachia* (arrow) in male gnathosoma and abdomen (combined Z sections). Right panels, bright field and fluorescence; left panels, fluorescence only.

*Wolbachia* localization was carried out in more than 50 adults taken from six ages (2 d, 4 d, 6 d, 8 d, 10 d and 13 d). However, we found that there was no age effect on *Wolbachia* localization. Generally, *Wolbachia* are located in the ovaries in the female abdomen and the gnathosoma (Fig. 3A and B). In male mites, although the signal specific to *Wolbachia* can be detected throughout the body, there is higher intensity in testes and gnathosoma (Fig. 3C and D).

**Figure 4:**
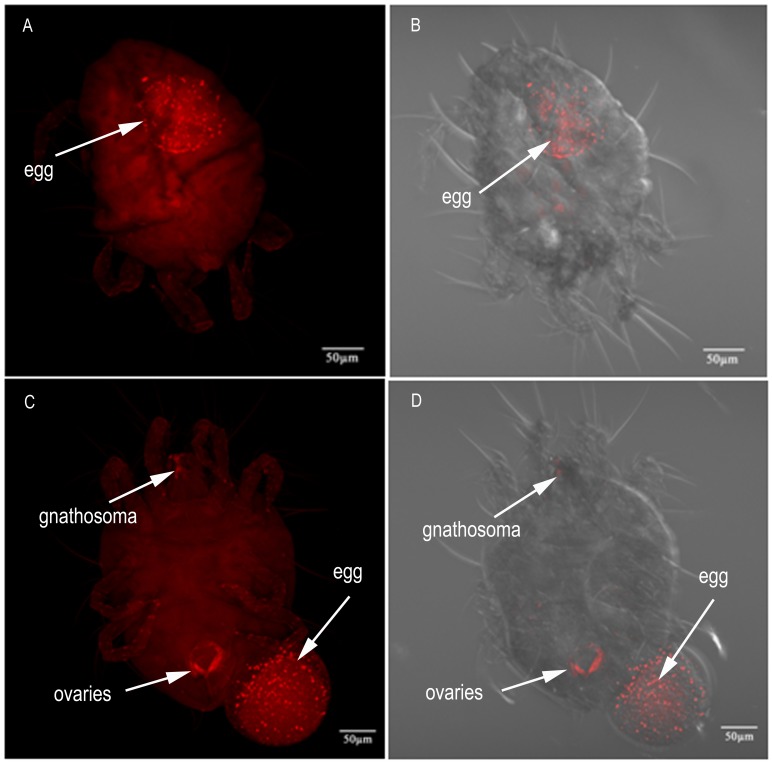
FISH of *T. urticae* females who were pregnant or were laying an egg. (A, B) *Wolbachia* (arrow) concentrated in the developing embryo in the female abdomen (combined Z sections). (C, D) *Wolbachia* (arrow) were transovarially transferred from the mother to the offspring through the egg (combined Z sections). Right panels, bright field and fluorescence; left panels, fluorescence only.

**Figure 5:**
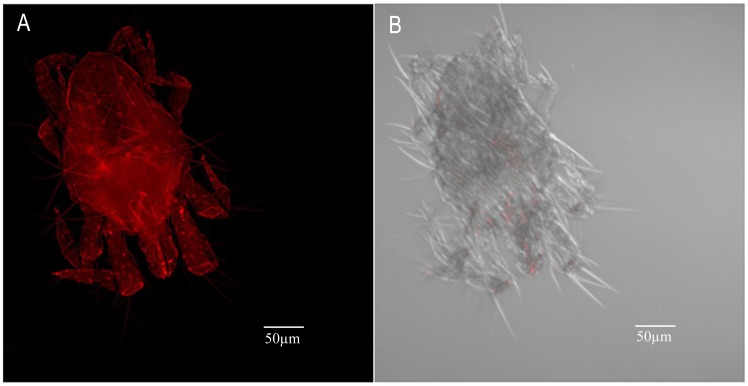
FISH of an uninfected *T. urticae* adult as a negative control. No *Wolbachia* specific signal was detected throughout the body. (A) fluorescence panel only (combined Z sections); (B) bright field and fluorescence panel (combined Z sections).

In pregnant females, *Wolbachia* signals were observed only in the developing embryo and were hardly detectable in other parts of the female body (Figure 4A–D), indicating that *Wolbachia* is ovarially transferred from the mother to the offspring through the egg. *Wolbachia* was also localized in egg-laying females (Fig. 4A and B). Interestingly, in our FISH analysis, most *Wolbachia* were transferred into the egg. At this stage, *Wolbachia* were present only at low density and exhibited a weak signal intensity in ovaries and in the middle of the gnathosoma (Fig. 4C and D). No signals were observed in the control (Fig. 5).

## Discussion

*Wolbachia* have attracted increasing attention over the past decade because an array of effects on their hosts may be used as a gene-driving system for genetic control of insect and other arthropod vectors and pests [37], [38], [39]. However, factors that can influence the densities of *Wolbachia* are rarely considered in laboratory studies, making the study of the interaction between *Wolbachia* and host unidirectional. In this study, we explored the relationships between *Wolbachia* and the spider mite *T. urticae* from a bidirectional perspective. We investigated the effects of *Wolbachia* on host reproduction, fitness, mating behavior (male mating competitiveness), as well as the effects of mating patterns on the density dynamics of *Wolbachia* during adult aging. Inbreeding effects on fitness traits differ between isofemale lines. As a result, it can be challenging to determine whether fitness differences are induced by *Wolbachia* or by the nuclear background [40]. In order to avoid inbreeding effects, each of the lines (infected line and uninfected line) used in this research was composed of more than 3 isofemale lines. We observed the fitness traits on different host backgrounds, and the effects were repeatable between infected and uninfected lines. Therefore, the fitness differences between infected and uninfected lines were induced by *Wolbachia* infection not by inbreeding effects.

*Wolbachia* infecting this spider mite population cause strong CI. CI is expressed as a significant reduction in egg hatchability and sex ratio (%females) in crosses between uninfected females and infected males. Interestingly, there is a strong reduction in the number of daughters in incompatible crosses, whereas male production was not significantly different in the compatible crosses. This pattern suggests that fertilized eggs, which would normally develop into females, have a higher mortality, and is concordant with the female mortality type of CI [9], [41], [42], [43]. However, this CI-inducing *Wolbachia* strain has no effect on the longevity of the female mites. In previous studies, Xie et al. reported both positive and negative effects of *Wolbachia* infection on longevity of *T. urticae* in China [26]. Similarly, both *Wolbachia*-associated benefits and costs in survival were found in *Drosophila melanogaster* [44], [45]. The different effects of *Wolbachia* on hosts have been attributed to several factors, including *Wolbachia* strain [46] and the nuclear background [47]. From an evolutionary perspective, bacteria and their hosts represent components of a dynamic interacting system that can evolve rapidly over time, which can explain the inconsistent effects of *Wolbachia* on host fitness detected in the above studies [15].

Several studies have investigated the relationship between *Wolbachia* and host mating behavior. Due to their effects on levels of pre- and postmating reproductive incompatibility, cytoplasmic-incompatible *Wolbachia* have been proposed to promote host speciation. The study by Koukou *et al* shows that the presence of *Wolbachia* (or another undetected bacterial associate) contribute to the level of pre-mating isolation between *Drosophila melanogaster* populations, which could be relevant to some speciation events [22]. In *Drosophila paulistorum*, there is a quantitative shift of *Wolbachia* densities from extremely low in native hosts to intermediate in interstrain hybrids, strongly suggesting that the causative agent of incipient *D. paulistorum* speciation is *Wolbachia* [48]. Furthermore, *Wolbachia* have strong effects on premating isolation between *D. paulistorum* semispecies in a titer-dependent manner. These results indicate that *Wolbachia* have the potential to trigger pre- and postmating isolation. In the present study, we found that different host mating patterns induced different *Wolbachia* density dynamics during host aging. However, no differences were observed between uninfected and infected males with respect to mating competitiveness. In order to reveal whether this cytoplasmic-incompatible *Wolbachia* plays a significant role in driving natural speciation of *T. urticae*, additional studies are needed to determine how the presence of *Wolbachia* alters mate behavior of *T. urticae*.

Our FISH results reveal that *Wolbachia* are more abundant in the reproductive tissues in both male and female *T. urticae*. Clark *et al* [49] proposed that *Wolbachia* infection in spermatocytes and then spermatids during sperm development is required for CI expression. *Wolbachia* induce high levels of CI only when spermatocytes or spermatids harbor this microbe. *Wolbachia* infection of somatic cyst cells, although sometimes present at high levels, does not result in significant CI expression. In addition, the study of Veneti *et al* revealed a strong positive correlation between CI expression levels and the percentage of infected sperm cysts [50]. On the basis of these theories, together with our FISH results and cross experiment results, we confer that the high level of CI expression in this *T. urticae* population results from the high percentage of infected sperm cysts and the presence of *Wolbachia* within the spermatocytes and spermatids of *T. urticae*. For *Wolbachia* to become fixed in a population, the bacterial cells must be transmitted to the next generation through the germline. It is important that *Wolbachia* were detected in the eggs in pregnant and egg-laying females, which suggests vertical transmission of *Wolbachia*. Until recently, it was unknown how *Wolbachia* enter the germline. Frydman *et al* answered this question by showing that *Wolbachia* accumulate in the somatic stem cell niche (SSCN) of *Drosophila germarium*, which is the site of egg chamber formation [51]. Further studies are needed to determine whether *Wolbachia* exhibits somatic stem cell niche tropism in *T*. *urticae*. Interestingly, *Wolbachia* densities are high in eggs present in pregnant mites. Meanwhile, the density measurement results indicate that *Wolbachia* density increases concomitantly with days after female emergence. These observations suggest that *Wolbachia* densities in females increase with increasing egg production. However, why would *Wolbachia* reside in the gnathosoma of mites? Why is *Wolbachia* signal less clear in pregnant females? More work remains to be performed to resolve these questions. To our knowledge, this is the first report to describe the localization of *Wolbachia* in spider mite adults.

Whereas our understanding of the *Wolbachia*-associated effects on hosts is rapidly expanding, our knowledge of the host factors that mediate *Wolbachia* dynamics is rudimentary. In this research, we reveal that host mating pattern affects *Wolbachia* density dynamics during host aging. Specifically, mating of an uninfected mite of either sex with an infected mite attenuates the *Wolbachia* density in the infected mite. This is the first empirical evidence indicating that host mating pattern affects *Wolbachia* density dynamics. During copulation, reproductive tissues probably experience high metabolic activity. In addition, we found a tropism of *Wolbachia* for the reproductive tissue in this spider mite population. Therefore, *Wolbachia* can be affected directly by host mating pattern. This is another phenotypic consequence of the tropism of *Wolbachia* for the hosts’ reproductive tissue. Recent reports have shown that *Wolbachia* in host reproductive tissues mediate the host cellular processes, such as mitotic activity and programmed cell death, which suggests that host cells and *Wolbachia* interact [51], [52], [53]. Our finding that *Wolbachia* density in males began to decline earlier than in females and the previous finding that *Wolbachia* replication is dependent on host cell replication [54] suggest that male host cells undergo earlier senescence and death than female host cells.

Our findings provide new avenues by which *Wolbachia* interacts with the host, indicating an urgent need to reveal the nature of the interaction between *Wolbachia* and their hosts.
